# Rhizoremediation of petroleum hydrocarbons: a model system for plant microbiome manipulation

**DOI:** 10.1111/1751-7915.13303

**Published:** 2018-07-31

**Authors:** Sara Correa‐García, Pranav Pande, Armand Séguin, Marc St‐Arnaud, Etienne Yergeau

**Affiliations:** ^1^ Centre INRS‐Institut Armand‐Frappier Institut national de la recherche scientifique Université du Québec Laval QC Canada; ^2^ Laurentian Forest Center Natural Ressources Canada Québec City QC Canada; ^3^ Institut de recherche en biologie végétale Université de Montréal and Jardin Botanique de Montréal Montréal QC Canada

## Abstract

Phytoremediation is a green and sustainable alternative to physico‐chemical methods for contaminated soil remediation. One of the flavours of phytoremediation is rhizoremediation, where plant roots stimulate soil microbes to degrade organic contaminants. This approach is particularly interesting as it takes advantage of naturally evolved interaction mechanisms between plant and microorganisms and often results in a complete mineralization of the contaminants (i.e. transformation to water and CO_2_). However, many biotic and abiotic factors influence the outcome of this interaction, resulting in variable efficiency of the remediation process. The difficulty to predict precisely the timeframe associated with rhizoremediation leads to low adoption rates of this green technology. Here, we review recent literature related to rhizoremediation, with a particular focus on soil organisms. We then expand on the potential of rhizoremediation to be a model plant‐microbe interaction system for microbiome manipulation studies.

## Introduction

Phytoremediation is the use of plants to remediate contaminated environments (usually soils, but also water). Many processes can be involved in the removal of the pollutants such as phytovolatilization (the removal of volatile compounds through plant tissues), phytotransformation (the transformation of contaminants from one state to another), phytostabilization (the stabilization of mobile contaminants in the soil), phytoextraction (the removal of trace elements from the soil and its fixation in plant tissues). Although all these processes involve both the plant and its microbiota, rhizoremediation clearly stands out as an integrated plant‐microbes endeavour. Rhizoremediation is the degradation of organic pollutants in the soil zone surrounding the plant roots (the rhizosphere), usually as a result of the stimulation of the catalytic activities of microorganisms by the plant roots (Pilon‐Smits, 2005). For many organic contaminants, such as most petroleum hydrocarbons, rhizoremediation results in the complete mineralization of the contaminants, effectively removing it from the environment.

**Figure 1:**
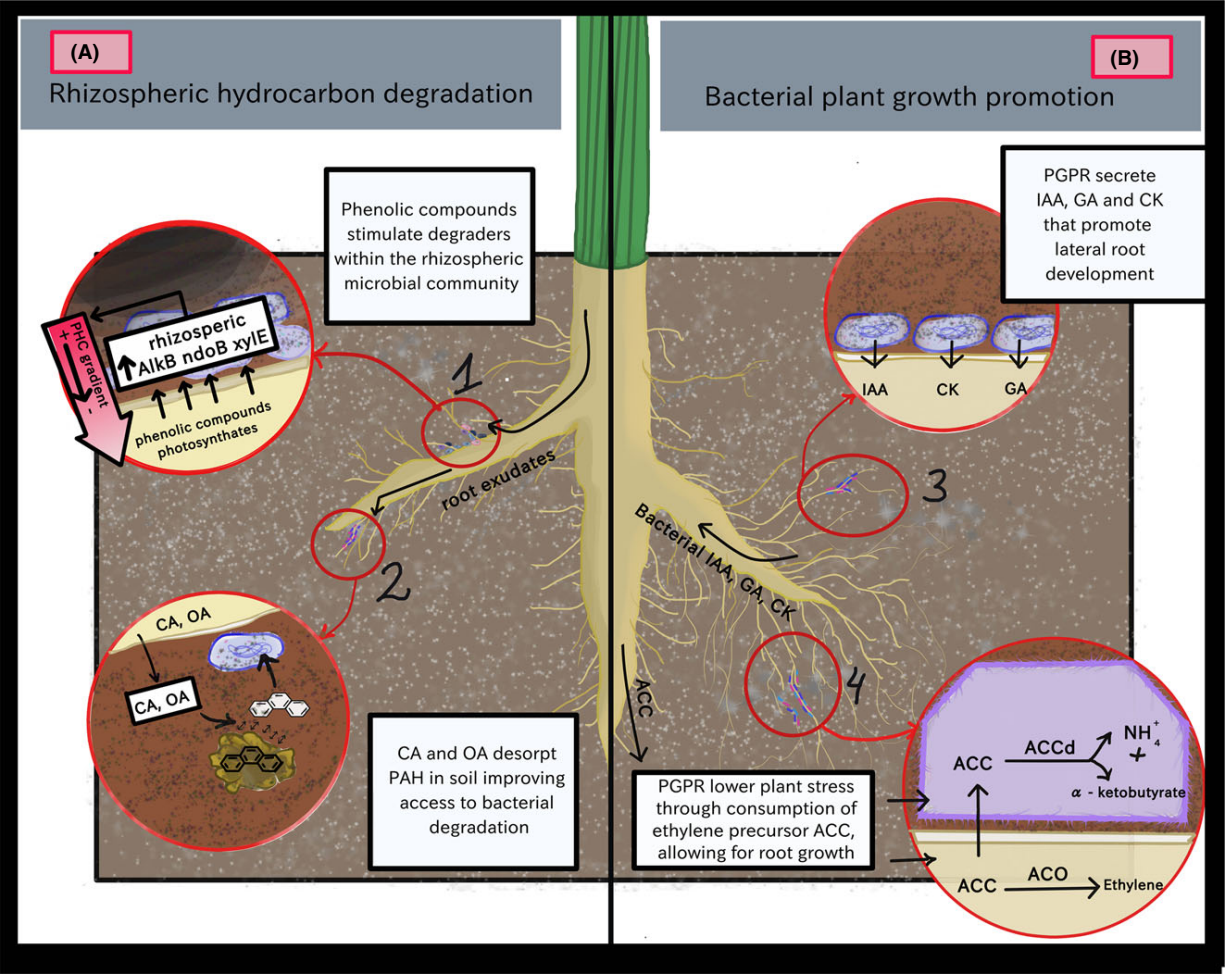
Major plant–microbe interactions occurring during rhizoremediation. In (A), plant root exudates (1) stimulate hydrocarbon‐degrading bacteria and (2) help to desorb contaminants attached to soil particles, making them more available to rhizobacteria. In (B), rhizosphere microorganisms promote plant growth through, among many other mechanisms, (3) the production of plant hormones and (4) the degradation of 1‐aminocyclopropane‐1‐carboxylic acid (ACC), the precursor of the stress hormone ethylene. PHC, petroleum hydrocarbons; *alkB*, alkane mono‐oxygenase, *ndoB*, naphthalene dioxygenase, *xylE*, catechol‐2,3‐dioxygenase; OA, oxalic acid; CA, citric acid; PAH, polycyclic aromatic hydrocarbon; PGPR, plant growth promoting rhizobacteria; IAA, indolacetic acid; CK, cytokinin; GA, gibberellic acid; ACC, 1‐aminocyclopropane‐1‐carboxylic acid; ACCd,ACC deaminase; ACO,ACC oxidase.

The principle behind rhizoremediation is simple: as the plant roots colonize the contaminated soil, as for any soil, they associate with a subset of the microorganisms present in the soil and stimulate them through the exudation of a variety of organic compounds (Kuiper *et al*., 2004) (Fig. 1A). Some of the microbes stimulated by the root exudates are also able to degrade petroleum hydrocarbons. Many facets of the rhizosphere environment make this soil zone particularly appropriate for the degradation of organic contaminants. First, the plant secondary metabolites that are part of the exudates are often structurally very similar to organic contaminants (Singer *et al*., 2003). This results in a heightened presence and activity of microbes being able to degrade organic contaminants in the rhizosphere, even in the absence of contaminants (Yergeau *et al*., 2014). Second, because of the presence of the root exudates, the rhizosphere microbial communities are generally more active and more abundant than microbial communities in the bulk soil (i.e. not under the influence of the roots) (Smalla *et al*., 2001; Kowalchuk *et al*., 2002). Third, the rhizosphere is generally recognized as a hotspot for horizontal gene transfer (Van Elsas and Bailey, 2002), and plasmids were shown to help microorganisms adapt to contamination stress and degrade organic compounds (Top and Springael, 2003; Sentchilo *et al*., 2013). Additionally, some root exudates help detach organic contaminants from the organic matter present in soil, making them more available to microbes (Gao *et al*., 2010). Altogether, this again highlights the distinct roles of plants and microorganisms during rhizoremediation: the plant act as a promoter for microbial degraders, by providing them with a suitable environment and stimulating them through root exudates. The suitability of the rhizosphere environment for microbial processes related to the degradation of hydrocarbons also exposes one of the major pitfalls of rhizoremediation: it only works where plant roots are. Therefore, in compacted or very clayey soils, or in cases where contamination is deeper than the root zone, or at too high concentration for roots to survive, rhizoremediation is not effective. As root growth patterns and exudates amount and quality differ between different plants, even between closely related genotypes (O'Toole and Bland, 1987; Jones *et al*., 2004; Manschadi *et al*., 2006), the choice of an appropriate plant genotype is crucial in rhizoremediation.

The rhizosphere microbes, especially bacteria, are thought to be the major players in organic contaminant degradation during rhizoremediation (Bell *et al*., 2014a,b; El Amrani *et al*., 2015), and recent plant‐microbe metatranscriptomic studies confirmed that the hydrocarbon degradation genes expressed in the root‐rhizosphere environment were mostly linked to bacteria (Gonzalez *et al*., 2018; Yergeau *et al*., 2018) (Box 1). Petroleum hydrocarbon contamination is often composed of a mixture of saturated aliphatic (alkanes) and aromatic hydrocarbons (including polycyclic aromatic hydrocarbons, PAHs). Microorganisms can degrade virtually all the hydrocarbons present in petroleum through various pathways, although with different efficiencies. In addition to this central role, microbes also have another major role in rhizoremediation (Fig. 1B). Indeed, microbes known as plant growth promoting rhizobacteria (PGPR) are recognized to have the capacity to increase plant growth (Kloepper and Schroth, 1978), and the ones that can increase root growth are particularly interesting in the context of rhizoremediation. On top of their ability to promote the growth of plants through the production of plant hormones or the mobilization of nutrients, PGPR also have the capacity to reduce plant stress through various mechanisms (Rajkumar *et al*., 2012; De Zelicourt *et al*., 2013), including through the reduction of ethylene concentrations in the roots (Glick *et al*., 1998; Glick, 2003) which would allow a plant to grow in highly contaminated environments without the adverse effects of stress (Burd *et al*., 2000).

Box 1 **The holobiont and the hologenome.**

All multicellular eukaryotes are associated with a wide diversity of microorganisms, forming an inseparable entity known as a holobiont (Rosenberg *et al*., 2009; Bordenstein *et al*., 2015; Van Opstal and Bordenstein, 2015; Theis *et al*., 2016). This observation has led Ilana Zilber‐Rosenberg and Eugene Rosenberg to enounce the hologenome theory of evolution that states that the hologenome (the combined genomes of the host and its microbiota) forms one of the units of evolution (Zilber‐Rosenberg and Rosenberg, 2008). Consequently, it is predicted that holobionts can rapidly evolve/adapt through their microbiota by: (i) horizontal gene transfer among their existing microbiota, (ii) recruitment of new microbes from the environment, (iii) shifts in the relative abundance/gene expression of various members of the microbiota. These mechanisms are thought to enable holobionts to adapt within a single or a few generations (Voss *et al*., 2015; Rosenberg and Zilber‐Rosenberg, 2016). It has recently been shown that the response of willows to stressful conditions (soil contamination) results in large shifts in the metatranscriptome of root and rhizosphere bacterial and fungal communities, but not in the plant root transcriptome (Gonzalez *et al*., 2018; Yergeau *et al*., 2018). Taken together, these results emphasize the importance of the plant microbiota in the response to environmental stresses and confirms that microbiota manipulation is a viable alternative to optimize phytoremediation (El Amrani *et al*., 2015; Quiza *et al*., 2015).

Rhizoremediation offers a unique system to study plant‐microbe interactions and experiment with microbiome manipulation approaches. First, the response variable of interest is easily measurable: a lowered soil contamination. Second, the hydrocarbon degradation pathways are well known, and the genes are well represented and annotated in databases (e.g. the biocatalysis/biodegradation database, http://eawag-bbd.ethz.ch/). Third, the capacity to degrade hydrocarbons is widespread among bacteria, and major players, such as *Pseudomonas* and *Rhodococcus* can be easily cultured. It is thus relatively easy to create consortia of hydrocarbon‐degrading bacteria, follow their fate in the environment using molecular tools and measure their effect on rhizoremediation efficiency. It is also possible to measure the effects of various manipulations on the hydrocarbon‐degrading microbiota abundance and activities in the rhizosphere using relatively inexpensive molecular tools such as qPCR.

## Soil organisms

### Microbes as hydrocarbon degraders

Hydrocarbon contamination is often a complex mixture of chemicals, requiring several different genes and pathways for its complete degradation. Most components can be classified as saturated aliphatic (alkanes) or aromatic hydrocarbons. Hydroxylation of an alkyl group catalyzed by oxygenases is usually the first step in the degradation of organic compounds alkanes. There are several categories of alkyl‐group hydroxylases, including cytochrome P450s (CYP) and alkane hydroxylase (Harayama *et al*., 1992). The alkane hydroxylase catalyzes the hydroxylation of the terminal carbon of alkanes and consists of three different subunits, including the membrane‐bound hydroxylase subunit encoded by *alkB*. CYP153, an enzyme of the CYP superfamily, can also catalyze the hydroxylation of alkanes (Van Beilen *et al*., 2006). Aromatic rings also need to be hydroxylated to be degraded, but the key step in aromatic hydrocarbon degradation is the opening of the hydroxylated aromatic ring, which is catalyzed by aromatic‐ring‐cleavage dioxygenases (Harayama *et al*., 1992). There are three main types of aromatic‐ring cleavage dioxygenases (intradiol, extradiol and gentisate/homogentisate) that can be differentiated based on their substrate and on the position where the ring fission occurs relative to the hydroxyl groups (Harayama *et al*., 1992).

Many specific bacteria of the phylum *Actinobacteria* and *Proteobacteria* were shown to degrade aliphatic and polycyclic aromatic hydrocarbons (PAHs). These bacteria are ubiquitous even in the most pristine environments (Yergeau *et al*., 2012, 2015a,b), probably because many of the petroleum hydrocarbon contaminants are widespread naturally occurring molecules. Recent metatranscriptomic studies in the rhizosphere highlighted several key taxa that responded to petroleum hydrocarbon contamination. For instance, in a pot study, transcripts related to *Alphaproteobacteria*,* Betaproteobacteria*,* Gammaproteobacteria* and *Acidobacteria* were more abundant in the rhizosphere of willows growing in contaminated soil as compared to non‐contaminated soils (Yergeau *et al*., 2014). Also, functional genes related to aromatic and aliphatic hydrocarbon degradation were more prevalent in the rhizosphere of willows growing in contaminated soils (Yergeau *et al*., 2014), and these genes were shown to be related to bacterial orders such as *Actinomycetales*,* Rhodospirillales*,* Burkholderiales*,* Alteromonadales*,* Solirubrobacterales*,* Caulobacterales* and *Rhizobiales* (Pagé *et al*., 2015). In the field, the differences in the expression of hydrocarbon degradation genes and in the active taxa between the rhizosphere of willows growing in contaminated and non‐con‐taminated soils varied and depended on the willow species (Yergeau *et al*., 2018). The abundance of PAH degrading genes was higher in phenanthrene‐contaminated soils planted with ryegrass as compared to non‐planted soils, with plants favouring the activities of bacterial degraders belonging to the *Pseudomonadales, Actinobacteria, Caulobacterales, Rhizobiales and Xhantomonadales* (Thomas and Cébron, 2016). Similarly, the presence of ryegrass was shown to stimulate the expression of bacterial PAH‐ring hydroxylating dioxygenase genes, such as *nidA3*,* pdoA*,* nahAc* and *phnAc* (Guo *et al*., 2017a,b). Several *Lotus corniculatus* (common bird's‐foot trefoil) and *Oenothera biennis* (common evening primrose) root endophytes belonging to the genera *Rhizobium*,* Pseudomonas*,* Stenotrophomonas* and *Rhodococcus* harbored genes encoding for CYP153 alkane hydroxylases and showed the capacity to grow with n‐hexadecane as sole source of carbon (Pawlik *et al*., 2017). Other plants, such as *Achillea millefolium* (yarrow)*, Soligado canadensis* (Canadian goldenrod)*, Trifolium aureum* (hop clover) and *Dactylis glomerata* (orchard grass)*,* growing in a heavily contaminated site, harbored hydrocarbon‐degrading bacterial endophytes mostly belonging to the *Actinobacteria* (Lumactud *et al*., 2016).

Fungi are particularly interesting in the context of rhizoremediation in view of their ability to form intimate associations with plant roots and colonize large volumes of soil through hyphal growth, and their production of a wide spectrum of extracellular hydrolytic enzymes, allowing them to grow on a wide variety of substrates. Ectomycorrhizal and saprotrophic basidiomycetes (white and brown rot fungi) have shown remarkable *in vitro* capacity for the degradation of PAHs and phenolic compounds, among others. Fungal hydrocarbon degradation is mostly an extracellular process, consisting in the release in the environment of active broad‐specificity oxidoreductase enzymes, such as laccases, manganese peroxidases and lignin peroxidases (Harms *et al*., 2011). In nature, these enzymes are mainly used to degrade lignin (a cross‐linked phenolic polymer), but their low specificity also allows them to degrade other phenolic compounds, such as the ones found in petroleum hydrocarbons (Karigar and Rao, 2011). A detailed list of fungi degrading organic pollutants is given in the review by Kadri *et al*. (2017), but it is still difficult to pinpoint which fungi is the most effective for rhizoremediation and what environmental factors influence this efficiency. For instance, the colonization of the rhizosphere and roots by fungi depend on factors like root exudate patterns, which are influenced by the presence of contaminant (Harms *et al*., 2011).

However, even if the microbial partners have the genetic capacity to degrade the contaminants, many substances are recalcitrant and elude microbial degradation. For instance, high molecular weight petroleum molecules (HMW) present a low bioavailability (Gamerdinger *et al*., 1997) and a high degree of adsorption to soil organic matter (Reddy and Sethunathan 1983) and are thus difficult to access by microbes. At the same time, high concentrations of these substances inhibit root elongation and ramification (Peña‐Castro *et al*., 2006; Ma *et al*., 2010). Furthermore, the efficiency of the rhizoremediation depends on the microbes capable of surviving within the polluted niche, which in turn depends on the microbial diversity originally present in the polluted soil.

### Microbes as plant‐growth promoters

One of the most important factor for maximizing the success of rhizoremediation is facilitating the growth of the plant root system. Rhizoremediation can only occur in the direct vicinity of plant roots and any treatment that can increase the growth of plant roots will result in a positive outcome for rhizoremediation (Fig. 1B). The root‐associated PGPR that can stimulate plant growth and help them tolerate stress, are frequently used under agricultural settings, but their use for phytoremediation has received less attention, representing a huge untapped potential.

Several mechanisms of action of PGPR have been described up to now (Olanrewaju *et al*., 2017). Some strains produce phytohormones related to plant growth and development such as indole‐3‐acetic acid (IAA) (Patten and Glick, 1996; Spaepen and Vanderleyden, 2011; Raut *et al*., 2017) and gibberellic acid (Gutiérrez‐Mañero *et al*., 2001). Other strains, such as phosphate‐solubilizers, mobilize nutrients from soil, making them more available for the plant (Rodrıguez and Fraga, 1999), whereas other PGPR compete with pathogens and suppress their action (Beneduzi *et al*., 2012). One particularly interesting way through which PGPR contribute to rhizoremediation is the suppression of the plant responses to stress, making the plant function as if there was no stress. Many mechanisms were shown to be involved in the enhancement of plant stress tolerance by microbes. Some target the modulation of plant stress genes (Timmusk and Wagner, 1999) or the reduction of the stress hormone ethylene levels through degradation of its precursor 1‐aminocyclopropane‐1‐carboxylic acid (ACC) by the bacterial enzyme ACC deaminase (Glick *et al*., 1998; Mayak *et al*., 2004; Glick, 2005). By reducing the levels of ethylene, bacteria harboring the ACC deaminase gene allow plants to function as if they were not subjected to stress and to develop their root system normally. In other cases, the expression of plant genes related to stress protection can be activated by bacterial volatile organic compounds (Ryu *et al*., 2004) or the drought stress‐related DNA methylation patterns can be modulated by endophytes (Hubbard *et al*., 2014).

Some studies have inoculated PGPR strains in the rhizosphere to improve plant growth under contaminated conditions. Liu *et al*. (2014) found that inoculating tall fescue with the ACC deaminase‐producing *Klebsiella* sp. enhanced plant growth and petroleum hydrocarbon remediation efficiency. Similarly, *Avena sativa* (common oat) growing in oil‐contaminated soil and inoculated with an *Acinetobacter* PGPR strain showed an increased dry mass and stem height compared to controls, along with a higher rate of decontamination (Xun *et al*., 2015). Inoculating bacterial strains transformed with a plasmid containing the ACC deaminase gene was shown to be more effective in promoting *Brassica napus* (rapeseed or canola) growth in PAH contaminated soils than when inoculating with the wild‐type strains (Reed and Glick, 2005). More recently, *Zea mays* growing in soils contaminated with 10 g kg^−1^ light crude oil and inoculated with a *Bacillus subtilis* strain producing ACC deaminase showed a significant increase in height, root length and biomass, which resulted in a 43% increase in petroleum hydrocarbon degradation compared to uninoculated plants after 60 days (Asghar *et al*., 2017). In a 3‐year field test carried at a site located in Ontario (Canada), the inoculation of PGPR resulted in an increased plant biomass and a reduction of the recalcitrant petroleum hydrocarbon fractions with HMW (Gurska *et al*., 2009). The inoculation of plant‐growth promoting endophytes was also shown to be effective for the rhizoremediation of petroleum hydrocarbons. The inoculation of the endophyte *Pseudomonas putida* PD1 in two willow clones (*Salix purpurea* 94006 and *Salix discolour* S‐365) growing in soil contaminated with 100 mg kg^−1^ phenanthrene resulted in a 25–40% increase in the degradation rate of this PAH compared to uninoculated controls (Khan *et al*., 2014).

Arbuscular mycorrhizal fungi (AMF) have multiple interesting roles that can improve rhizoremediation. For instance, under contaminated settings AMF can modify the rhizosphere microbial communities (Iffis *et al*., 2016, 2017) and improve plant growth (Xun *et al*., 2015), thereby likely increasing the efficiency of rhizoremediation. It is also well known that AMF can improve plant nutrition (Smith and Read, 2008) or provide protection against pathogens (Hamel, 2007; Tang *et al*., 2009), which could result in increased root growth and increased stimulation of the rhizosphere microorganisms. The AMF *Glomus mosseae* was shown to improve rhizoremediation of PAH, with significantly higher reductions in the concentrations of chrysene and dibenz(a,h)anthracene in AMF‐inoculated pots as compared to non‐inoculated pots (Joner *et al*., 2001). Interestingly, non‐inoculated pots had degradation levels similar to those observed for non‐planted pots. In a field study, the AMF associated with willows were strongly structured by the contamination levels, with reduced diversity at higher contamination levels, suggesting that only a narrow number of AMF can thrive in these highly contaminated environments (Hassan *et al*., 2014). Ectomycorrhizal fungi were also shown to be influenced by contamination levels, with some species only associating with local willow genotypes under high contamination levels (Bell *et al*., 2014). The same fungus was subsequently shown to be related to willow Zn uptake at a metal contaminated field site (Bell *et al*., 2015). A holistic approach combining AMF and PGPR inoculation, as it has already been proposed in the context of crop production (Nadeem *et al*., 2014), could lead to more effective rhizoremediation strategies due to the synergistic effect these organisms can have in improving plant physiology and by increasing of the volume of soil under the influence of the roots.

### Other soil organisms

Although not part of the plant microbiome *per se*, many other organisms living in the soil can influence the interactions between the plant, the microbiome and contaminated soils during rhizoremediation. As such, they could be interesting targets for plant microbiome manipulation. These organisms include nematodes, protists, collembola, and earthworms among others. In this section, we will focus on earthworms as a model soil organism for modulating rhizoremediation. Earthworms are typical soil inhabitants making up > 80% of the biomass of soil macrofauna (Yasmin and D'Souza, 2010), and are frequently found in the rhizosphere environment (Springett and Gray, 1997). Earthworms can survive in the most highly contaminated soils as only water soluble compounds can be absorbed through their skin (Jager, 1998), which excludes most toxic PAHs (Ma *et al*., 1998) and PCBs (Beyer and Stafford, 1993). For instance, Zavala‐Cruz *et al*. (2013) recorded the presence of *Pontoscolex corethrurus*,* Gossodrillus* sp. and *Dichogaster salines* in a site polluted with crude oil for 20 years with petroleum hydrocarbons concentrations up to 12 000 mg kg^−1^. The model species *Eisenia fetida* can survive to up to 3500 mg kg^−1^ of petroleum hydrocarbons (Geissen *et al*., 2008). Several studies have shown that the presence of earthworms improves or accelerates the degradation rate of several PHC. For instance, the application of the earthworm *E. fetida* resulted in the removal of 92% of anthracene from an arable soil after 56 days, as compared to 57% in the untreated soil (Delgado‐Balbuena *et al*., 2016).

Earthworm are also having a strong impact on soil microbial community composition (Emmerling and Paulsch, 2001), and the bacterial taxa containing major hydrocarbon degraders (such as the *Proteobacteria*) are often more abundant when earthworms are present. For instance, the application of the earthworms to an anthracene contaminated soil resulted in a shift in the soil microbial community with a decrease in the relative abundance of *Gemmatimonadetes*,* Chloroflexi* and *Acidobacteria* and an increase in the *Proteobacteria* compared to the untreated soil (Delgado‐Balbuena *et al*., 2016). Similarly, the *Alpha*‐ and *Betaproteobacteria* were shown to be mostly unaffected after their passage through the digestive system of the earthworms (Nechitaylo *et al*., 2010). *Betaproteobacteria* were in fact stimulated in the presence of the earthworm *P. corethrurus* (Bernard *et al*., 2012). Additionally, the degradation of many hydrocarbon substances may start in the direct environment of the earthworm, as many known degraders such as *Rhodococcus* and *Azotobacter* were found in the burrows of *Lumbricus terrestris* (Tiunov and Dobrovolskaya, 2002), whereas other known degraders such as *Pseudomonas*,* Alcaligenes, Acidobacterium*, and the fungus *Penicillium*, were found in the intestine and cast of earthworms (Singleton *et al*., 2003).

In addition to the ones mentioned above, earthworms have other roles that could make them a key component of rhizoremediation. Indeed, earthworms are recognized ecological engineers, contributing to the mineralization and humifaction of organic matter (Lavelle and Spain, 2001), being highly mobile vectors moving bacteria in and out the rhizosphere (Luepromchai *et al*., 2002), and improving water infiltration and soil aeration (Bartlett *et al*., 2010). Although all these activities are expected to positively stimulate rhizoremediation, only a few studies have tested the effect of earthworms in the context of rhizoremediation. One such study looked at PCB rhizoremediation and found that ryegrass co‐inoculated with AMF and earthworms decreased soil PCB contents by 79.5% as compared to 74.3% for AMF alone, 62.6% for earthworms alone or 58.4% for ryegrass alone (Lu *et al*., 2014). Earthworms and other soil fauna thus represent an unexploited potential for rhizoremediation of petroleum hydrocarbons.

## Plant root exudates

Root exudates have been identified as a major ecological driver that actively modulate the microbial community composition, diversity and activity of the rhizosphere (Fig. 1A). The plant exudes a variety of specialized antimicrobials and signalling molecules (e.g. flavonoids, salicylic acid and phytoalexins), carbon (e.g. organic acids, aromatic compounds) and nitrogen (e.g. amino acids) compounds. Therefore, only a specific group of microbes that can utilize these compounds are selectively enriched in this highly competitive environment (Gomes *et al*., 2003; Haichar *et al*., 2008; Berg and Smalla, 2009). However, exudation is not for the sole benefit of microbes; it also directly benefits the plant itself. For instance, organic acids, such as malate, citrate and oxalate are often present in the rhizosphere, and in addition to being a carbon source for many microbes, they are involved in many plant processes like nutrient acquisition, metal detoxification, and alleviation of stress (Jones, 1998). Plants confronted with stressful environments normally respond by increasing root exudation (Jones *et al*., 2004; Qin *et al*., 2007), which leads to increased microbial biomass (Esperschütz *et al*., 2009) and activity (Yergeau *et al*., 2014) in the rhizosphere. Root exudates can also improve the availability of contaminant for microbial degradation, as it was shown that the desorption of phenanthrene and pyrene from soil particles was increased by the addition of citric and oxalic acid (Gao *et al*., 2010). An increasing amount of scientific evidence points towards the crucial importance of exudates as mediators of hydrocarbon rhizoremediation (Martin *et al*., 2014; Rohrbacher and St‐Arnaud, 2016).

Interestingly, many compounds found in the rhizosphere are analogous to organic contaminants (Singer *et al*., 2003), including terpenes, lignin derived components and flavonoids (Hartmann *et al*., 2009). Negative correlations between the concentrations of plant root exudates and petroleum hydrocarbons have been observed, with lower concentrations of PHC observed close to the roots where maximum concentrations of exudates are found (Gao *et al*., 2011; Ling *et al*., 2013), because exudates induce the degradation of PHC by rhizospheric microorganisms (Sun *et al*., 2010). For instance, the phenolic root exudates fomorin, caffeic acid and protocatechuic acid were linked to bacterial degradation of tricyclic and tetracyclic PAHs in the rhizosphere (Ely and Smets, 2017) and increases in phenolic root exudates have been associated with higher rates of degradation of benzo[a]pyrene in the rhizosphere of *Phragmites australis* (cosmopolitan common reed) (Toyama *et al*., 2011). In fact, the rhizosphere of plants are often enriched in microbial genes related to the degradation of organic contaminants that are actively expressed even in the absence of contaminants (Yergeau *et al*., 2014). Conversely, in the absence of plants, supplementing a PAH contaminated agricultural soil and a pyrene‐spiked soil with maize and soybean exudates resulted in an increased initial PAH degradation that faded through time as exudates were depleted (Guo *et al*., 2017a,b). The interaction between *Mycobacterium* and the root exudates accelerated the removal of PAH by provoking a shift in the soil bacterial community structure and diversity (Guo *et al*., 2017a,b).

## Rhizoremediation as a model for microbiome manipulation

### Choosing the right plant

Choosing the right plant is crucial to achieve optimal rhizoremediation. The main aspects to take into account when choosing the best fit for rhizoremediation are root morphology, plant tolerance to the contaminant and root exudate profile. For instance, various *Poaceae* species (grasses) are often selected for rhizoremediation purposes as they produce a dense secondary root system that can harbor an abundant microbial community (Adam and Duncan, 2002; Lee *et al*., 2008; Gaskin and Bentham, 2010; Barrutia *et al*., 2011). However, most *Poaceae* plants are not appropriate when the pollutants have reached deeper layers in the soil, and deeper rooting plants, such as trees like willows or poplars should be preferred in these cases (Kuzovkina and Volk, 2009). The quantity and quality of root exudates also vary substantially even between closely related plant genotypes, resulting in significant differences in the recruitment and stimulation of microbes in the rhizosphere (Lundberg *et al*., 2012; Yergeau *et al*., 2018). This variation in the microbiome then results in different degradation rates in the rhizosphere of different plant genotypes. The choice of the right plant is thus one of the actionable ways to manipulate the rhizosphere microbiome and increase remediation rates.

Many studies have shown that different plant species have different capacity for the rhizoremediation of petroleum hydrocarbons. For instance, the removal rate of eight PAHs (tetracyclic and pentacyclic) was measured in the rhizosphere of *Echinacea purpurea* (purple coneflower*)*,* Festuca arundinacea* (tall fescue), and *Medicago sativa* (alfalfa) growing in pots (Liu *et al*., 2015). Although the degradation rates increased for all plants as compared to the unplanted controls, the level of degradation strongly varied by plant species (Liu *et al*., 2015). Similarly, in a field study on a former coal mine site, the capacity for various legume tree species [*Cassia siamea* (cassod tree), *Albizia lebbeck* (lebbeck)*, Delonix regia* (flame tree) and *Dalbergia sissoo* (North Indian rosewood)] to reduce soil PAH levels was evaluated (Mukhopadhyay *et al*., 2017). The results showed that the degradation rates varied from 51.5% to 81.6% among the trees tested (Mukhopadhyay *et al*., 2017).

The response of the plant itself to contamination will also have a determining effect on the success of rhizoremediation. Willow genotypes showed large differences in the response of their growth patterns and physiology to contamination (Grenier *et al*., 2015). These results were mirrored in the transcriptomic response of the rhizosphere microbiota (Yergeau *et al*., 2018), with the willow species showing the largest decreases in biomass and photosynthetic capacity also showing the largest decreases in the expression of genes in their associated microbiota. This suggests that the physiological responses of the willow genotypes to contamination could be good indicators of their rhizoremediation potential. Additionally, when growing in highly contaminated soils in Canada, North American willow genotypes showed a strong association with an ectomycorrhizal fungus, *Sphaerosporella brunnea*, whereas Asian and European genotypes did not associate with this particular fungus (Bell *et al*., 2014a,b). Therefore, the region of origin of the plant appears to have an importance, with local plants being better adapted to interact with the local beneficial soil microbiota.

### Modifying the microbiota

**Figure 2:**
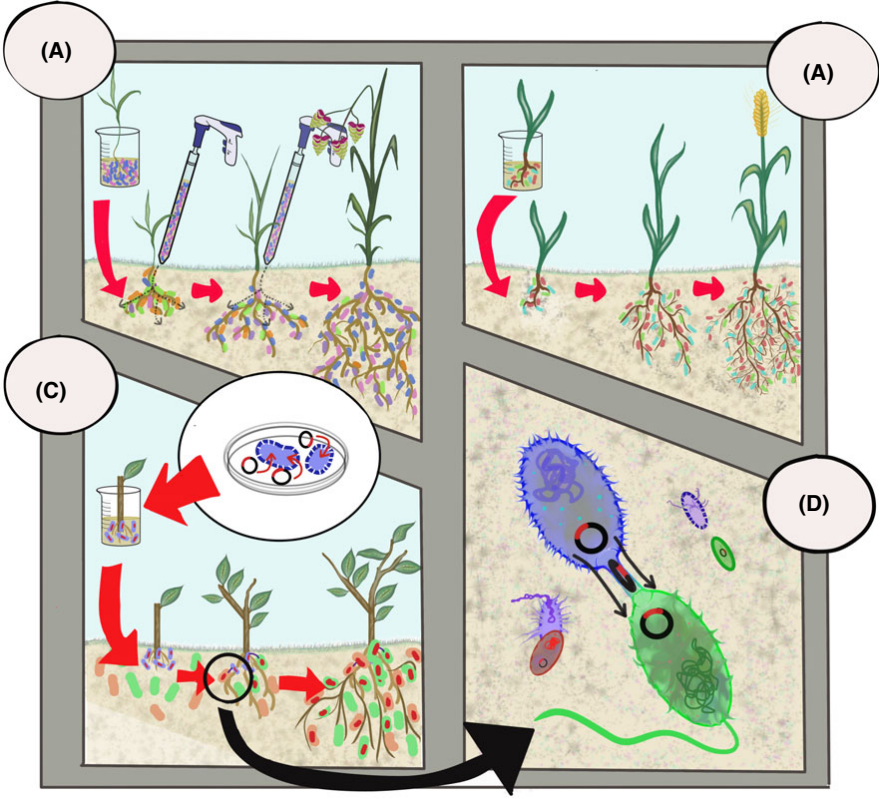
Examples of promising plant microbiome manipulation approaches for enhanced rhizoremediation: (A) repeated inoculation of a consortia of hydrocarbon‐degrading microorganisms, (B) early inoculation of a plant growth promoting rhizobacteria (PGPR) consortia, (C and D) inoculation of a bacteria harboring hydrocarbon degradation or plant growth promotion genes on a plasmid.

Generally, indigenous hydrocarbon‐degrading microbes in contaminated soils can be efficiently stimulated by plants (Yergeau *et al*., 2014; Pagé *et al*., 2015) or fertilizers (Yergeau *et al*., 2009, 2012; Bell *et al*., 2011). Bioaugmentation (inoculations) with a single or a few hydrocarbon‐degrading strains is thus generally ineffective (Thomassin‐Lacroix *et al*., 2002), and it has been shown that pre‐selecting microorganisms that can degrade hydrocarbons results in less efficient degradation than using the entirety of the microbes present in a soil (Bell *et al*., 2016). However, a recent report suggested that the success of invasion by the inoculated microbes could be increased by successive inoculation (Fig. 2), as the initial inoculation opens up a niche space for the invader (Mallon *et al*., 2018). The inoculum density could also play a role for single strain inoculations, as the inoculation of *Lolium perenne* (perennial ryegrass) with different concentrations of the alkane degrading *Pantoea* sp., resulted in maximum plant growth, diesel degradation, bacterial abundance and CYP153 alkane hydroxylase gene expression in the treatment with the densest inoculation (10^8^ cell cm^−3^ soil) (Shabir *et al*., 2016). In addition, several studies have reported successful single PGPR strain inoculations in the context of rhizoremediation (Reed and Glick, 2005; Gurska *et al*., 2009; Xun *et al*., 2015; Asghar *et al*., 2017).

Alternatively, the inoculation of consortia is generally more efficient than individual strains for degrading hydrocarbons (Ghazali *et al*., 2004; Heinaru *et al*., 2005; Jacques *et al*., 2008; Mrozik and Piotrowska‐Seget, 2010) (Fig. 2). Some of the desired properties for the microbial constituents of a rhizoremediation consortia have been suggested: (i) be proficient in the colonization of the plant root surface in the rhizosphere, (ii) be able to survive, grow and not outcompete the rest of the members of the consortia, (iii) be able to attach to the root surface, (iv) be able to promote plant growth or the growth of other members of the consortia, (v) be able to handle abiotic stress, especially contaminant stress, (vi) be able to grow to the desired density under stressful conditions (Yang *et al*., 2009; Calvo *et al*., 2014). Consequently, candidate microbes for a rhizoremediation consortia should at least be selected for the strength of their association with the plant, with traits such as a strong chemotaxis towards plant root exudates and a strong attachment to the plant root surface (Yang *et al*., 2009; Bashan *et al*., 2014).

Taking the concept of consortia one step further, some studies have tried using synthetic communities for rhizoremediation (Pizarro‐Tobias *et al*., 2015). These synthetic communities should be designed to improve positive interactions, like commensalism and cooperation, while preventing negative interactions like predation and parasitism. Furthermore, several hydrocarbon degraders should be combined to ensure the presence of a diverse and redundant hydrocarbon degradation gene pool. Ideally, synthetic communities should be prepared from hundreds to thousands of strains, making it nearly impossible to test all interactions between consortia members. It is also difficult to maintain synthetic microbial communities for several generations (Johns *et al*., 2016), probably because of the numerous interactions happening simultaneously between members of the synthetic community (Stubbendieck *et al*., 2016). The use of naturally occurring, highly performing communities could be a better alternative, removing the need to test the compatibility of isolated strains with each other. Indeed, exposing willows to differentially selected initial soil communities can result in large differences in biomass when willows are grown under high stress levels, and these differences persist through time even though rhizosphere microbial communities become eventually identical (Yergeau *et al*., 2015a). This lends weight to the idea that exposing the plant partner to a different complex microbiota during its establishment can result in an improved growth in contaminated soils. However, many technical hurdles are facing the propagation and inoculation of complex microbiota at large scales.

Inoculating bacteria that harbored hydrocarbon degradation genes on mobile genetic elements has resulted in some successes (Fig. 2). For example, Weyens *et al*. (2009) observed a decrease in trichloroethylene (TCE) evapotranspiration (thus an increased degradation) after inoculation of hybrid poplars with a *Pseudomonas* strain containing a plasmid coding for the constitutive expression of the TCE degradation genes. This strain altered the rhizosphere community, even though it did not establish in this compartment. The strain did establish inside plant tissues, and the plasmid it contained was transmitted to other members of the endophytic community (Weyens *et al*., 2009). Similarly, the inoculation of two *Burkholderia* strains containing a plasmid coding for the constitutive expression of toluene degradation genes resulted in an improved plant growth and an increased toluene degradation (reduced evapotranspiration) (Taghavi *et al*., 2005). Although the two strains could not be detected in the plants, the plasmid they carried was detected in various other endophytic bacteria (Taghavi *et al*., 2005). Both these examples used endophytic bacteria, but a similar approach could be used for rhizoremediation, especially in view of the enhanced horizontal gene transfer rates in the rhizosphere (Van Elsas and Bailey, 2002).

### Predictive models

Because it is a biological process, the time for soil decontamination by way of phytoremediation is difficult to estimate accurately, which often makes this option less attractive. Recent work has provided interesting evidence that various ecosystem processes could be predicted from microbiological data. For instance, the degradation of diesel in arctic soils could be predicted by the initial bacterial diversity and the abundance of specific assemblages of *Betaproteobacteria*, which was also related to the soil organic matter content (Bell *et al*., 2013). In that study, high *Betaproteobacteria* abundance was positively correlated with high diesel degradation. The predictability with which bacterial communities respond to these disturbances suggest that costly and time‐consuming chemical assessments of contaminated sites may not be necessary in the future and could be replaced by simple biological assessments (quantification of *Betaproteobacteria*). Similarly, the growth of willows after 100 days in highly contaminated soil could be predicted by the initial bacterial and fungal community composition and the initial relative abundance of specific taxa (Yergeau *et al*., 2015a). The Zn accumulation by willows growing for 16 months in a former landfill could be predicted by the relative abundance of specific fungal taxa in the rhizosphere after 4 months of growth (Bell *et al*., 2015). It therefore appears that the composition and relative abundance of the early colonizers of the plant environment are good predictors of its future behaviour. Creating predictive models could assist in choosing the right plant and the right microorganisms for a specific site without the need for labor‐intensive and costly preliminary trials, and, more importantly, estimate more precisely the time that will be needed for complete rhizoremediation.

## Perspectives

Despite the remarkable advances detailed above, phytoremediation remains a marginal option for *in situ* soil remediation (Mench *et al*., 2010). The major obstacle to market penetration is that many sites to be decontaminated are in peri‐urban areas and need to be efficiently decontaminated over a short period, which is incompatible with the current practice of *in situ* phytoremediation. Additionally, phytoremediation is rarely suggested as a remediation technique by accredited experts because it is believed to be inefficient and because of the inability to precisely determine the duration of this biological process as it depends on contaminant and soil natures, plant used, environmental conditions and microbial activities (Montpetit and Lachapelle, 2015, 2016). One of the main reasons behind this was the low level of knowledge shown by accredited experts in the field of soil remediation partly due to poor communication from scientists (Montpetit and Lachapelle, 2015, 2016). Therefore, on top of research efforts aiming at better understanding the plant–microbe interactions during rhizoremediation, future endeavours should also (i) set‐up large scale demonstration experiments, potentially using integrated bioremediation approaches (Megharaj and Naidu, 2017), (ii) partner with environmental consulting firms and accredited experts, (iii) develop a genomics‐based tool to suggest management strategies and predict the duration of phytoremediation and (iv) test novel microbiome management approaches applicable at the field scale, such as inocula combining PGPR and microbial degraders (Baez‐Rogelio *et al*., 2017).

## Conflict of interest

None declared.
